# Nicotinamide mononucleotide (NMN) protects bEnd.3 cells against H_2_O_2_‐induced damage via NAMPT and the NF‐κB p65 signalling pathway

**DOI:** 10.1002/2211-5463.13067

**Published:** 2021-02-09

**Authors:** Xiujun Deng, Xinghuan Liang, Haiyan Yang, Zhenxing Huang, Xuemei Huang, Chunfeng Liang, Yaqi Kuang, Yingfen Qin, Faquan Lin, Zuojie Luo

**Affiliations:** ^1^ Department of Laboratory The First Affiliated Hospital of Guangxi Medical University Nanning China; ^2^ Department of Endocrinology The First Affiliated Hospital of Guangxi Medical University Nanning China; ^3^ Department of Blood transfusion The First Affiliated Hospital of Guangxi Medical University Nanning China

**Keywords:** brain microvascular endothelial cells, inflammatory pathway, nicotinamide mononucleotide, nicotinamide phosphoribosyltransferase, oxidative stress

## Abstract

An increasing number of studies have shown that nicotinamide mononucleotide (NMN) can inhibit not only ageing but also oxidative stress and inflammatory reactions by improving energy metabolism. However, the role of NMN in regulating the anti‐apoptotic, antioxidative stress and inflammatory responses of brain microvascular endothelial cells is still unknown. Therefore, here we studied the effects of NMN on H_2_O_2_‐induced oxidative damage of bEnd.3 cells. In this study, we found that NMN could inhibit the NF‐κBp65 inflammatory signalling pathway and increase the expression of the enzymes NAMPT, VEGF and eNOS, alleviating H_2_O_2_‐induced apoptosis in bEnd.3 cells. Taken together, these results suggest that NMN reduces H_2_O_2_‐induced oxidative stress and apoptosis and improves cell functions by inhibiting the NF‐κBp65 inflammatory pathway and increasing NAMPT expression.

## Graphical abstract

Nicotinamide mononucleotide (NMN) has antioxidant and anti‐apoptosis effects in diabetes and cerebrovascular disease models. We verified that NMN can alleviate the apoptosis of bEnd.3 cells induced by oxidative stress, and we further found that the anti‐oxidation and anti‐apoptosis effects of NMN were related to the regulation of NF‐KBp65 pathway and NAMPT enzyme.

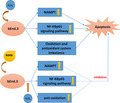

**Abbreviations**
BBB: blood–brain barrier
bEnd.3: mouse brain endothelial cells
CAT: catalase
CCK‐8: Cell Counting Kit‐8
DMEM: Dulbecco’s modified Eagle’s medium
eNOS: endothelial nitric oxide synthase
ICAM‐1: intercellular adhesion molecule
IL‐1: interleukin‐1
LDH: lactate dehydrogenase
MMP: matrix metalloproteinase
NAD^+^: nicotinamide adenine dinucleotide
NAMPT: nicotinamide phosphoribosyltransferase
NF‐κB p65: nuclear factor kappa B p65
NMN: nicotinamide mononucleotide
ROS: reactive oxygen species
SOD: superoxide dismutase
TNF: tumour necrosis factor‐α
VEGF: vascular endothelial growth factor

Diabetes is a common disease. At present, the prevalence and incidence of diabetes have risen sharply worldwide.

Compared with nondiabetic patients, diabetic patients are more prone to stroke, and it has been reported that haemorrhagic stroke in diabetic patients is approximately 1.5 times that in nondiabetic individuals [1]. During the natural development of diabetes mellitus, endothelial cell dysfunction and the relative dysfunction of various endothelial mediators have generally been considered to be early signs of diabetic metabolic disorders. In turn, each metabolic disorder (insulin resistance, compensatory hyperinsulinaemia, hyperglycaemia, oxidative stress, excessive free fatty acid release and lipotoxicity) occurring in diabetes mellitus may affect endothelial functions [2]. In the brain, endothelial cells are a specialized monolayer of cells arranged on the wall of the vascular lumen and constitute the most critical portion of the physical barrier between the blood and the brain called the blood–brain barrier (BBB) [3]. In the central nervous system, the BBB plays an important physiological role in regulating paracellular permeability, ion balance, nutrient transport and cerebral haemodynamics, and the molecular characteristics of the BBB, adhesion junctions and transporters are particularly important [4, 5]. The neurodegenerative changes caused by diabetes, obesity and Alzheimer's disease have similar pathways, including overlapping pathways of oxidative stress, mitochondrial dysfunction and inflammation, accompanied by impaired integrity of the blood–brain barrier [6].

Oxidative stress and inflammation are the key mediators of blood–brain barrier dysfunction. Abnormal metabolism and systemic inflammation lead to the excessive production of ROS in the brain and the release of inflammatory factors, which further lead to imbalances in endothelial cell regulatory protease and dysfunctional endothelial cell secretion [7, 8]. Endothelial nitric oxide synthase (eNOs) and vascular endothelial growth factor (VEGF) are mainly involved in angiogenesis and changes in vascular permeability [9]. When vascular endothelial cells are affected by oxidative stress and other harmful factors, VEGF can activate many pathways, among which eNOS‐induced impairments in the NO pathway are characteristics of impaired vascular endothelial function, which leads to a decrease in vasodilation factors, the expression of inflammatory factors and a decrease in angiogenesis [10, 11]. A large amount of ROS produced by oxidative stress stimulate endothelial cells to express intercellular adhesion molecule (ICAM‐1) and secrete matrix metalloproteinase (MMP), which attract and bind lymphocytes and monocytes in blood vessels, leading to inflammation [12]. In addition, oxidative stress also involves the activation of the NF‐κB pathway, which controls the synthesis and release of the inflammatory factors interleukin‐1 (IL‐1) and tumour necrosis factor‐α (TNF‐α) [13] and further destroys the BBB.

NMN, as a direct enzymatic product of NAMPT, is a small molecule that can easily pass through the blood–brain barrier and mediate cell death, oxidative stress and inflammatory reactions under pathological conditions [14, 15]. In recent years, increasing evidence has shown that NMN exerts a protective effect on cerebrovascular diseases and neuronal damage related to diabetes, ageing and oxidative stress, and these pathological processes often involve the reduction or depletion of NAD^+^ [16, 17, 18]. Further studies showed that NMN has an anti‐inflammatory effect and improves endothelial dysfunction induced by IL‐1β or TNF‐α, and this process is related to the regulation of intracellular NAD^+^ levels by NMN in a manner dependent on the expression of CD78 on the surface of endothelial cells [19]. However, the impact of NMN on H_2_O_2_‐induced oxidative stress and inflammation in bEnd.3 cells and changes in nampt levels remain unclear. Therefore, the aim of the present study was to investigate whether NMN inhibits H_2_O_2_‐induced oxidative stress and inflammation in bEnd.3 cells and to determine the molecular mechanisms.

## Materials and methods

### Cell culture

bEnd.3 cells were purchased from Procell Life Science & Technology Co., Ltd. (Wuhan, China). bEnd.3 cells were maintained at 37°C in a humidified atmosphere of 5% CO_2_ and 95% air in DMEM containing 10% heat‐inactivated fetal bovine serum (Gibco, Grand Island, NY, USA), 50 U·mL^−1^ penicillin and 50 mg·mL^−1^ streptomycin (Solarbio, Beijing, China).

### Preparation and treatment with NMN

Nicotinamide mononucleotide was purchased from Sigma‐Aldrich (St. Louis, MO, USA). NMN was prepared as a stock solution in double‐distilled water and was diluted to the final concentration before the experiment. The prepared stock solution of 10mM NMN was stored at −20 °C.

### Cell viability assay

bEnd.3 cells were treated with different concentrations of H_2_O_2_ (0, 500, 700, 800, 900 and 1000 µm) and NMN (0, 100, 200, 300, 500 and 600 µm) for 24 h. Then, bEnd.3 cells were treated with 100 µL of DMEM and 10 µL of Cell Counting Kit‐8 Reagent (AbMole BioScience, Inc., Houston, TX, USA). The plates were incubated in the dark for 1 h at 37°C. The optical density (OD) value was measured using a Synergy H1 Reader (BioTek, Winooski, VT, USA) at 450 nm.

### Apoptosis analysis

bEnd.3 cells were collected by trypsinization, washed twice with cold PBS and then resuspended in 1x binding buffer, which is a buffer containing Ga^+^. Then, 100 µL of the solution was transferred to a 5‐ml culture tube before being incubated with 5 µL of Annexin V‐FITC and 5 µL of PI (BD Biosciences, Franklin Lakes, NJ, USA) in the dark for 25 min at room temperature. Stained cells were detected by flow cytometry (BD Biosciences) within an hour.

Apoptotic cells were also analysed by Hoechst 33258 staining. After the appropriate treatments, bEnd.3 cells were fixed with 4% paraformaldehyde for 20 min; then, the 4% paraformaldehyde was removed, the cells were rinsed with PBS three times, and Hoechst 33258 (Beyotime, Shanghai, China, 500 µL/well) was added and incubated for 20–30 min. The images were captured under a fluorescence microscope (Nikon Corporation, Tokyo, Japan). Apoptotic dying cells were identified as the cells with blue fragmented, condensed nuclei, and the percentage of apoptotic bEnd.3 cell was calculated as from total number of cell population. The percentage of apoptotic cells was analysed by Image J software.

### Measurement of ΔΨm

After experimental treatments, the cells were incubated with JC‐1 (Beyotime) staining solution at 37 °C for 20 min and then resuspended in 500 µL of precooled JC‐1 staining buffer. Fluorescence microscopy (Nikon Corporation, Tokyo, Japan) was used to obtain the photographs of the cells. The complexes with red fluorescent were formed in cells with normal mitochondria. In mitochondria‐damaged cells, JC‐1 maintained a monomeric form and showed green fluorescence. The ratio of red to green light in the obtained photographs was quantitatively analysed by ImageJ software.

### ROS level measurement

Treated cells were washed with PBS and incubated with serum‐free basal medium, which contained 2,7‐dichlorodihydrofluorescein diacetate (DCFH‐DA, Beyotime), for 20–30 min at 37°C in the dark. The cells were washed with serum‐free cell culture solution, and then, the fluorescence intensity in the cells, which represented ROS production, was measured by immunofluorescence microscopy (Nikon Corporation, Tokyo, Japan) at an excitation wavelength of 488 nm and an emission wavelength of 525 nm.

### Detection of catalase (CAT) and superoxide dismutase (SOD) activity

The activities of the antioxidant enzymes, catalase (CAT) and superoxide dismutase (SOD) were measured using the Catalase Assay Kit (Nanjing Jiancheng Bioengineering Research Institute, Nanjing, China) and Micro‐method SOD Activity Detection Kit (Solarbio, Beijing, China). Every 5 million cells were mixed with 1 mL PBS or superoxide dismutase lysis buffer, lysed by ultrasound and then centrifuged at 8000 ***g*** for 10 min at 4 °C, and the supernatant was taken for detection. The absorbance of CAT and SOD was measured on Synergy H1 Reader (BioTek) with wavelengths of 560nm and 405 nm, respectively, and the result was expressed as U·mg^−1^ protein.

### Determination of lactate dehydrogenase (LDH) and caspase‐3

The effect of NMN on cell cytotoxicity was determined using the Spectrophotometric LDH Determination Kit (Solarbio, Beijing, China). The cells were added 1 mL LDH extract to be sonicated (ultrasound for 3 s, 10‐s interval, repeated 30 times) and then centrifuged at 8000 ***g*** at 4 °C for 10 min, and the supernatant was measured on Synergy H1 Reader (BioTek) at 450nm absorbance. The result was expressed as U·mg^−1^ protein. In addition, Colorimetric Caspase‐3 Activity Assay Kit (Solarbio) was used to determine caspase‐3 activity. The cells were lysed by shaking with 200 µL caspase‐3 lysis solution, ice‐bathed for 10 min, shaken again and then centrifuged at 12 000 ***g*** at 4 °C for 10 min, and the supernatant was taken to determine the OD value at 405nm using Synergy H1 Reader (BioTek). The percentage increase in caspase activity was expressed by the following formula: 100%×[(experimental treatment group OD ‐ blank control OD)/(experimental control OD ‐ blank control OD)].

### Reverse transcription–quantitative polymerase chain reaction (RT–qPCR)

The RNA purity was determined after extracting the total RNA from cells using NucleoZOL Reagent (Macherey‐Nagel, Düren, Germany) according to the manufacturer’s protocols. Oligo‐dT primers and AMV reverse transcriptase (Takara, Shiga, Japan) were used to synthesize cDNA. Amplification of the cDNA was performed using the FastStart Universal SYBR Green Master Mix (Roche, Mannheim, Germany) on an ABI StepOnePlus (Applied Biosystems, Boston, MA, USA). The primer sequences used are listed in Table 1. GAPDH was used as an internal reference. The quantification of gene expression was performed using the 2^−ΔΔCt^ method.

**Table 1:** Sequences of the primers employed for reverse transcription–quantitative polymerase chain reaction

Name	Forward primer(5′−3′)	Reverse primer(5′−3′)
NF‐KBp65	TCGAGTCTCCATGCAGCTACGG	CGGTGGCGATCATCTGTGTCTG
Nampt	CGCAAGAGACTGCTGGCATAGG	ACTGTGCTCTGCTGCTGGAAC
IL‐1β	TCGCAGCAGCACATCAACAAGAG	TGCTCATGTCCTCATCCTGGAAGG
ICAM‐1	CTGAAAGATGAGCTCGAGAGT	AAACGAATACACGGTGATGGTA
MMP‐9	CAAAGACCTGAAAACCTCCAAC	GACTGCTTCTCTCCCATCATC
VEGF	TAGAGTACATCTTCAAGCCGTC	CTTTCTTTGGTCTGCATTCACA
ENOs	CTGAGAGCCTGCAATTACTACC	TTTCCACAGAGAGGATTGTAGC
TNF‐α	ATGTCTCAGCCTCTTCTCATTC	GCTTGTCACTCGAATTTTGAGA
GAPDH	TGCTGTCCTGTATGCCTCTG	TTGATGTCACGCACGATTTCC

### Western blot analysis

The cells were cultured with H_2_O_2_ (800 µm) and pretreated with NMN (300 µm) for 24h, and then, cells were lysed with radioimmunoprecipitation assay buffer (Solarbio) to obtain total protein extracts or used Nuclear and Cytoplasmic Extraction Kit (Shanghai Ya Enzyme Biotechnology Co., Ltd, Shanghai, China) to extract nuclear protein. Subsequently, the protein concentration was determined using a BCA Protein Analysis Kit (Beyotime Institute of Biotechnology). The proteins were separated by SDS/PAGE (Bio‐Rad, Hercules, CA, USA, USA) and then transferred onto a PVDF membrane (Millipore, Billerica, MA, USA), which was incubated with 5% nonfat dry milk TBST. The protein‐containing membrane was incubated with anti‐NF‐κB p65, anti‐IL‐1, anti‐TNF‐α, anti‐ICAM‐1, anti‐MMP9 (Cell Signaling Technology, Danvers, MA, USA, Cat. No. 3033, Cat. No. 12507, Cat. No. 11948, Cat. No. 67836, Cat. No. 13667), anti‐NAMPT (Abcam, Cambridge, UK, Cat. No. 236874), anti‐GAPDH and anti‐PCNA (Signalway Antibody, Nanjing, China, Cat. No. 21612, Cat. No. 29168) antibodies at 4 °C overnight. Then, the membrane was incubated with horseradish peroxidase‐conjugated secondary antibodies (Signalway Antibody, Cat. No. L3012) for 1 h at 37°C. Subsequently, the proteins were confirmed by visualization using a LI‐COR system (Odyssey, Lincoln, NE, USA) according to the manufacturer’s instructions. The intensity of the bands of interest was analysed with ImageJ software version 1.8.0.

### Statistical analysis

Graphs were generated using GraphPad Prism 6.0 (GraphPad Software Inc., San Diego, CA, USA) and ImageJ 1.8.0 (National Institutes of Health, USA). The results are expressed as the mean ± SD and were analysed with SPSS 17.0 software. One‐way ANOVA was selected to analyse the differences among the groups. A value of *P* < 0.05 was considered statistically significant.

## Results

### NMN protects cells from H_2_O_2_‐induced decline in proliferation activity

**Fig. 1:**
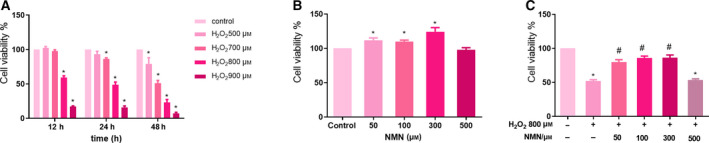
NMN alleviates decreased viability of bEnd.3 cells induced by H_2_O_2_. A‐B. bEnd.3 cells were exposed to different times (12, 24, 48 h) and various concentrations of H_2_O_2_ (0, 500, 700, 800, 900 µm) and NMN (50, 100, 300, 500 µm) for 24 h. C. NMN pretreated cells with 800 µm H_2_O_2_ to induce damage. Results are expressed as means ± SD and are representative of three independent experiments. Data were analysed by one‐way ANOVA with LSD’s test for multiple comparisons. *P < 0.05, vs. control group. #*P* < 0.05, vs. 800 µm H_2_O_2_ group.

To investigate the effects of NMN and H_2_O_2_ on the viability of bEnd.3 cells, a CCK‐8 Kit was used to measure cell proliferation rate. The results showed that the cells were exposed to 800 µm H2O2 for 24h, the survival rate of the cells decreased by about 50% compared with the control group, while 50, 100 and 300 µm NMN for 24 h significantly improved cell proliferation rate compared with the control group (Fig. 1A.B); furthermore, bEnd.3 cells were damaged by 800 µm H_2_O_2_ after pretreatment with 50, 100, 300 and 500 µm NMN for 24 h, and it was observed that 50–300 µm NMN significantly increased cell proliferation rate compared with the that of the H_2_O_2_ group (*P* < 0.05), and no significant differences were observed among the 50–300 µm NMN groups; however, the effect of 500 µm NMN on cell viability was not different from that of the H_2_O_2_ group (Fig. 1C).

### NMN reduces H_2_O_2_‐induced bEnd.3 cell apoptosis

**Fig. 2:**
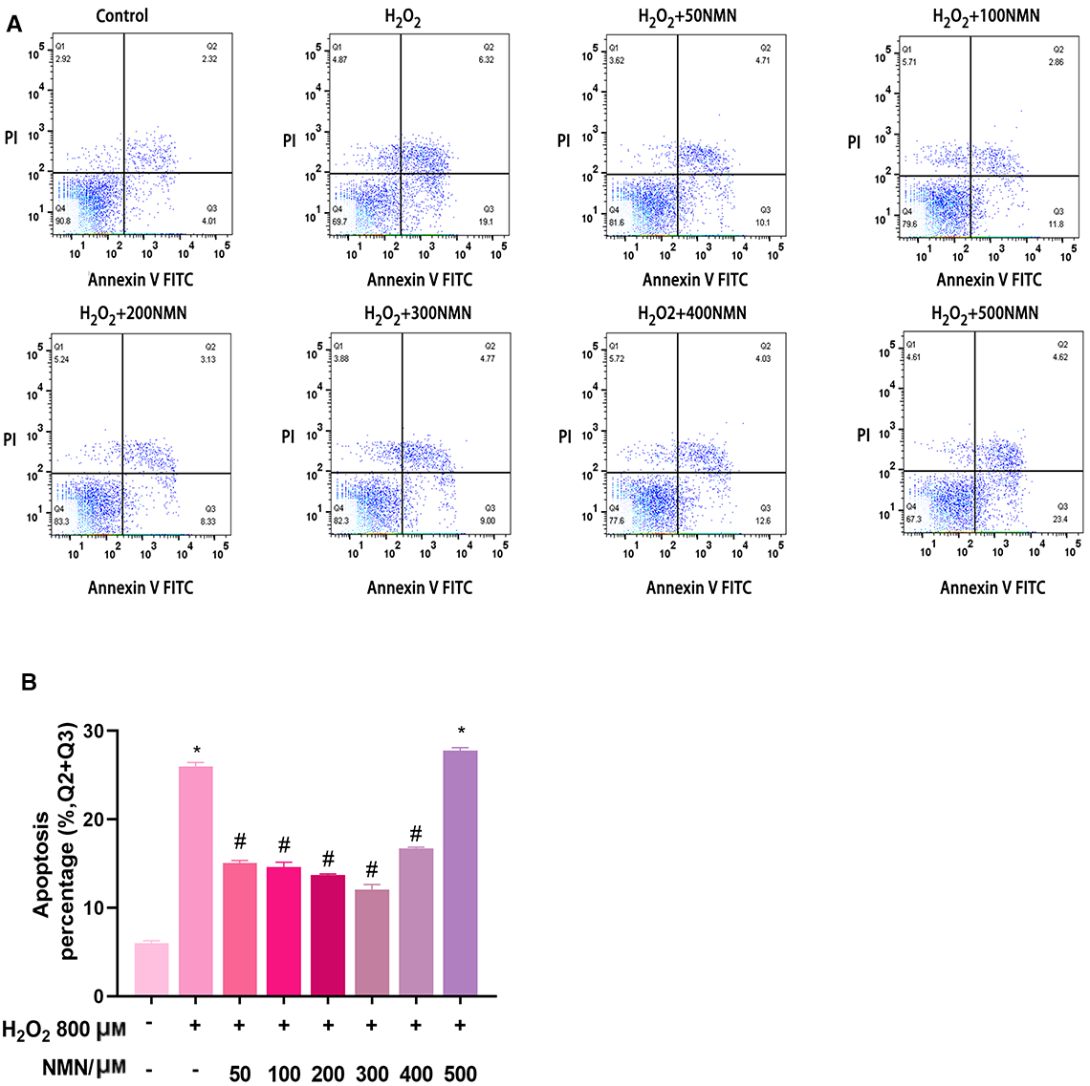
(A) Early and late apoptotic events in bEnd.3 cells injured by H_2_O_2_ and the effects of various concentrations of NMN on 800 µm H_2_O_2_‐induced apoptosis were detected by flow cytometry. The top left quadrant indicates nonapoptotic cells; the top right quadrant represents late apoptotic events; the bottom right quadrant represents early apoptotic cells; and the bottom left quadrant represents living cells. B. The apoptosis rate of bEnd.3 cells induced by H_2_O_2_ that were pretreated with different concentrations of NMN was analysed by flowjo software. Results are expressed as means ± SD and are representative of three independent experiments. Data were analysed by one‐way ANOVA with LSD’s test for multiple comparisons. *P < 0.05, #*P* < 0.05 vs. the control group. #*P* < 0.05 vs. the 800 µm H_2_O_2_ group.

To determine whether NMN improved cell viability by suppressing H_2_O_2_‐induced cell apoptosis in bEnd.3 cells, the rate of apoptosis was evaluated by flow cytometry. The results showed that after incubation with 800 µm H_2_O_2_ for 24 h, the apoptosis rate was increased compared with that of the control group, while NMN (50, 100, 200, 300 and 400 µm) reduced the apoptosis rate compared with that of the H_2_O_2_ group (*P* < 0.05); however, 500 µm NMN did not affect apoptosis (*P* > 0.05) (Fig. 2). These results suggest that the effect of NMN on bEnd.3 cell apoptosis in vitro was consistent with the results of the CCK‐8 assay.

**Fig. 3:**
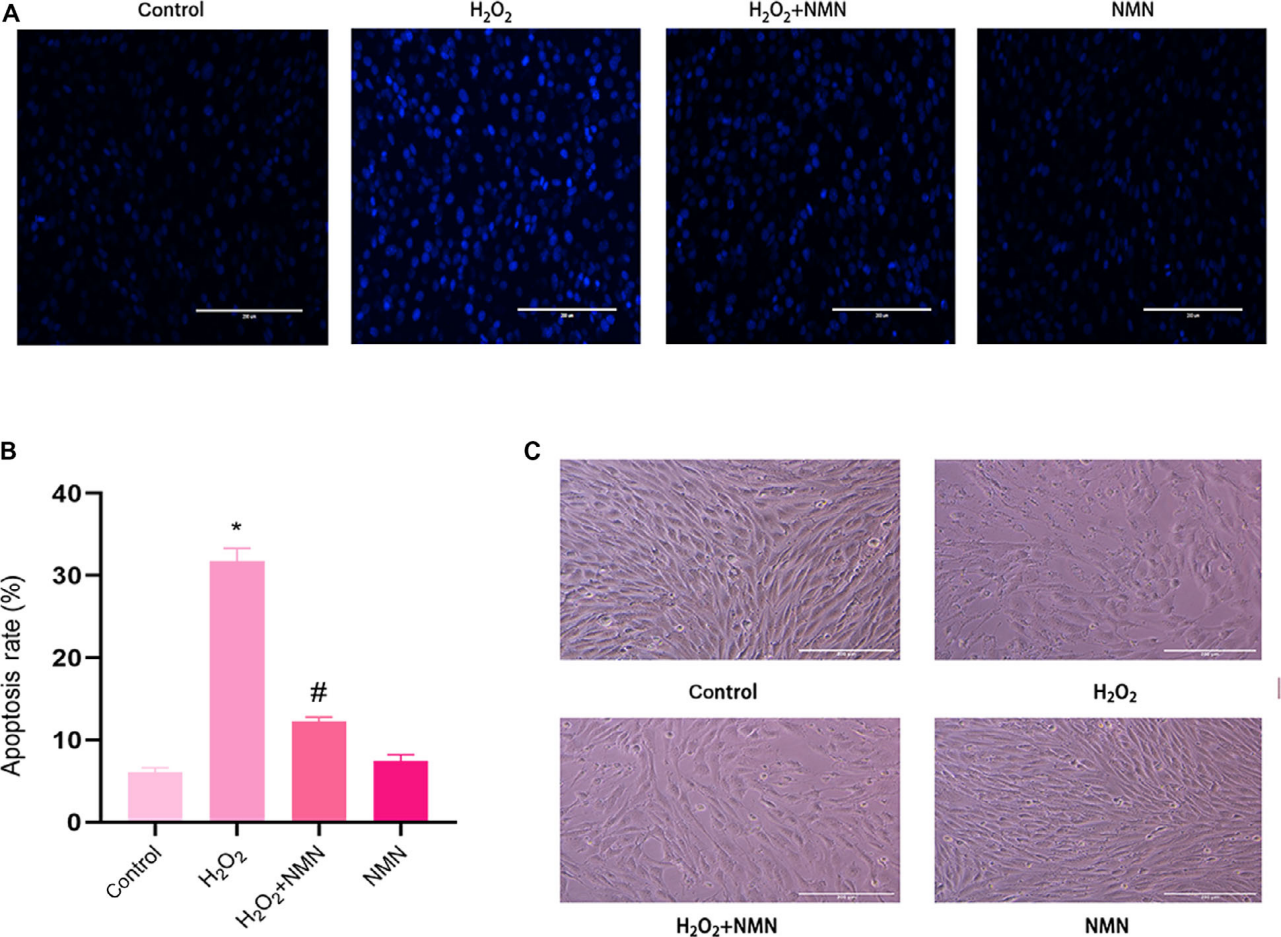
(A) Apoptosis of cells that induced by H_2_O_2_ (800 µm) and NMN (300 µm) reduced apoptosis induced by H_2_O_2_ was stained with Hoechst 33258. (B) The apoptotic and total cells were analysed by imagej software, and the apoptosis rate was presented as % of the total number of nuclei C. bEnd.3 cells were cultured with 800 µm H_2_O_2_ and 300 µm NMN (×10 scale bar = 100 μm). Results are expressed as means ± SD and are representative of three independent experiments. Data were analysed by one‐way ANOVA with LSD’s test for multiple comparisons. *P < 0.05, #*P* < 0.05 vs. control group. #*P* < 0.05, vs. 800 uM H_2_O_2_ group.

Hoechst 33258 staining was further used to show that NMN alleviated H_2_O_2_‐induced early apoptosis in bEnd.3 cells. After bEnd.3 cells were pretreated with 300 µm NMN for 24 h, the number of bright blue fluorescent cells was significantly reduced, indicating that NMN can significantly reduce the apoptotic cells and nuclear concentration caused by H_2_O_2_ (Fig. 3A,B). In addition, the cells in the H_2_O_2_ treatment group grew slowly, lost the appearance of paving stones, changed their morphology and were flat; the number of adherent cells decreased compared with that of the control group; and 300 µm NMN reversed these changes (Fig. 3C).

### Effect of H_2_O_2_ on the mitochondrial membrane potential of bEnd.3 cells

**Fig. 4:**
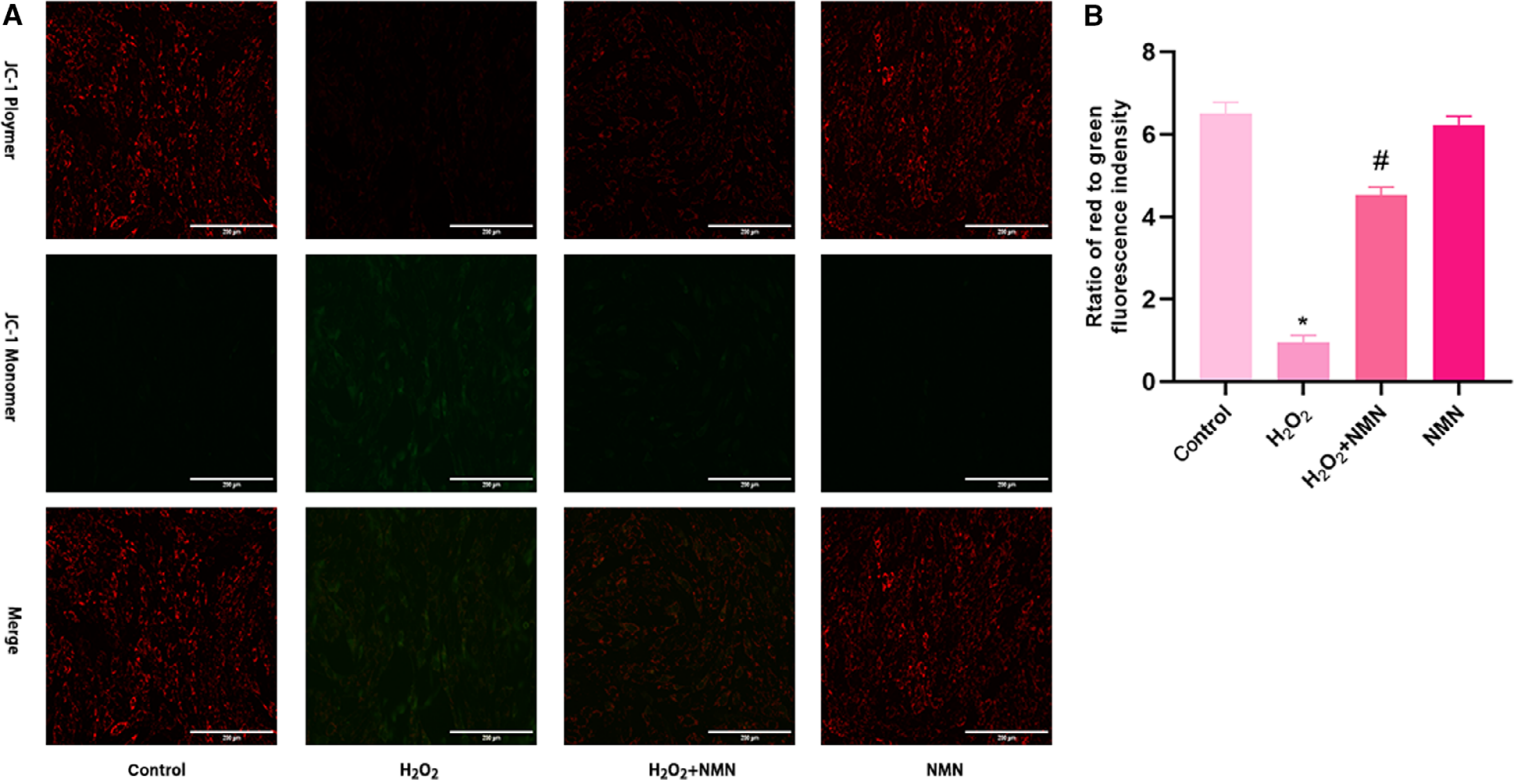
Protective effect of NMN (300 µm) on mitochondrial damage induced by H_2_O_2_ (800 µm). (A) Immunofluorescence microscopy of mitochondrial membrane potential shown by JC‐1 staining (×20; scale bar = 200 μm). (B) The ratio of red‐green fluorescence intensity indicating the mitochondrial membrane potential by JC‐1 staining. Results are expressed as means ± SD and are representative of three independent experiments. Data were analysed by one‐way ANOVA with LSD’s test for multiple comparisons. *P < 0.05 vs. the control group. #*P* < 0.05 vs. the H_2_O_2_ group.

Oxidative stress and inflammation‐induced decreases in mitochondrial membrane potential are markers of early cell apoptosis. Changes in mitochondrial membrane potential were determined by JC‐1 staining. The results showed that the red fluorescence of H_2_O_2_‐induced cells decreased, the green fluorescence increased, and the ratio of red‐to‐green fluorescence decreased significantly compared with those of the control group (Fig. 4A,B), indicating that mitochondrial membrane potential decreased. After NMN treatment, the red‐green fluorescence ratio increased, indicating that the mitochondrial membrane potential increased.

### Effect of NMN on ROS levels in bEnd.3 cells damaged by H_2_O_2_

**Fig. 5:**
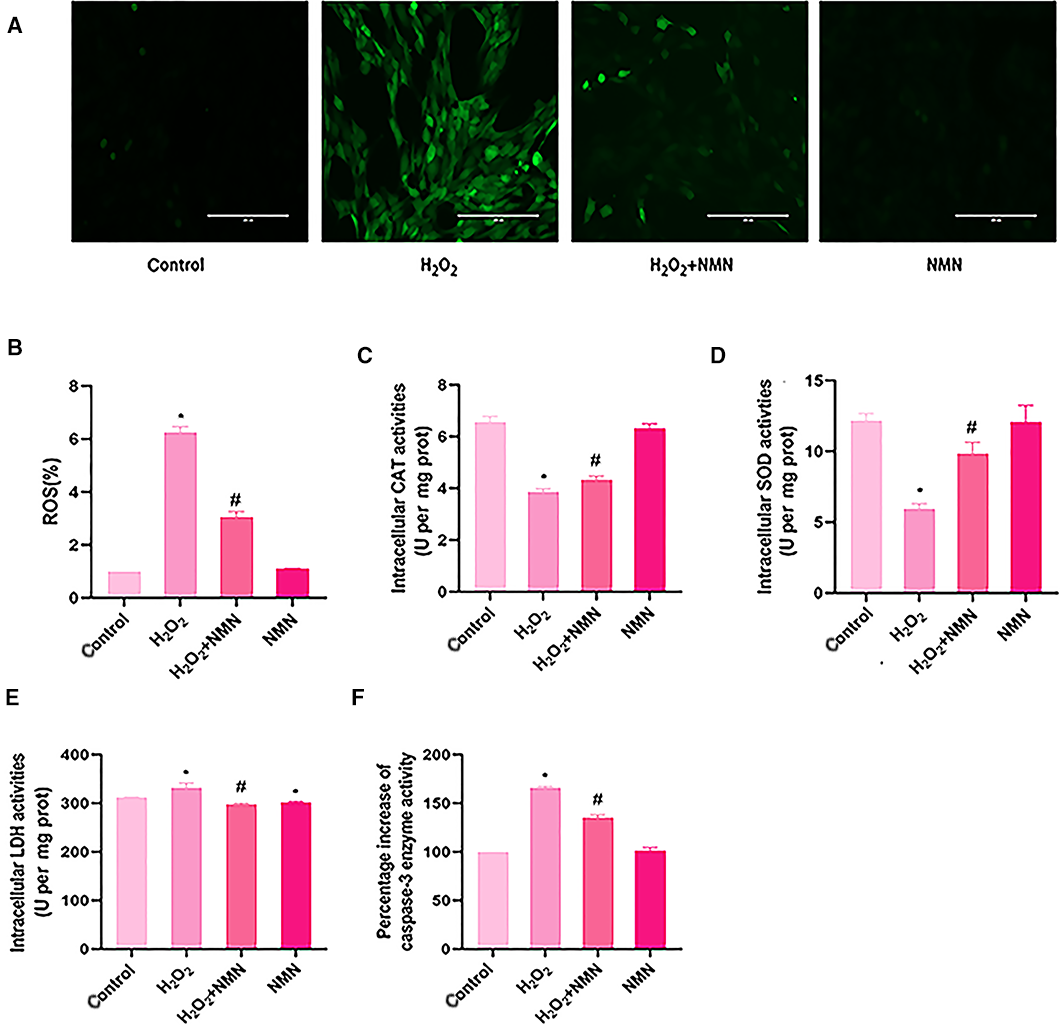
Nicotinamide mononucleotide decreased intracellular ROS, LDH and caspase‐3 level but increased CAT and SOD activities treated with H_2_O_2_. bEnd.3 cells were pretreated with 300 µm NMN for 24 h and then exposed to 800 µm H_2_O_2_. (A) ROS was measured by the fluorescent probe DCFH‐DA and imaged by fluorescence microscopy (×20; scale bar = 200 μm). (B) The average fluorescence intensity was calculated by ImageJ software and reflects the intracellular ROS level. C‐F. The activities of cellular CAT, SOD, LDH and caspase‐3. The CAT, SOD and LDH levels were normalized per milligram of protein. The level of caspase‐3 was expressed as the percentage increase in activity compared with the normal group. Results are expressed as means ± SD and are representative of three independent experiments. Data were analysed by one‐way ANOVA with LSD’s test for multiple comparisons. *P < 0.05, #*P* < 0.05 vs. the control group. #*P* < 0.05 vs. the H_2_O_2_ group.

The fluorescent probe DCFH‐DA can be used to detect reactive oxygen species; reactive oxygen species in cells can oxidize nonfluorescent DCFH to generate fluorescent DCF, and the fluorescence intensity represents the level of reactive oxygen species in cells. There were significant differences in the relative fluorescence intensities among the control group, oxidative damage group and NMN 300 µm group. Compared with that of the control group, the relative fluorescence intensity of the oxidative damage group was significantly enhanced, and a large amount of ROS was produced in the oxidative damage group. Compared with the oxidative damage group, the 300 µm NMN group exhibited significantly reduced ROS induced by H_2_O_2_. However, NMN alone had no effect on ROS levels in cells (Fig. 5A,B).

### NMN increases the activities of CAT and SOD in H_2_O_2_‐treated bEnd.3 cells

To investigate whether NMN mediates antioxidant enzyme activity to protect bEnd.3 cells from H_2_O_2_‐induced apoptosis, we measured the activities of CAT and SOD. When bEnd.3 cells were treated with 800 µm H_2_O_2_, the activities of SOD and CAT in the cells were significantly decreased compared with the control group, whereas pretreatment with 300 µm NMN significantly increased the activities of CAT and SOD compared with the H_2_O_2_ treatment group (*P* < 0.05) (Fig. 5C,D).

### NMN reduces the elevation of LDH and caspase‐3 in bEnd.3 cells under H_2_O_2_ conditions

After the cells were pretreated with NMN and then treated with H_2_O_2_ for 24h, the cytotoxicity was determined by measuring the level of LDH. The results showed that the cytotoxicity of the cells in the H_2_O_2_ treatment group increased compared with control group (*P* < 0.05) and the cell cytotoxicity decreased in H_2_O_2_ + NMN group compared with H_2_O_2_ group (*P* < 0.05) (Fig. 5E). In addition, we also measured the activity of caspase‐3, and the results showed that bEnd.3 cells treated with H_2_O_2_ significantly increased caspase‐3 compared with the control group, while caspase‐3 after NMN pretreatment significantly decreased compared with the H_2_O_2_ treatment group (*P* < 0.05) (Fig. 5F). The above showed that NMN inhibited H_2_O_2_‐induced cytotoxicity and caspase‐3 activation to reduce bEnd.3 cell apoptosis.

### Effect of H_2_O_2_ and NMN on the expression of genes associated with the NF‐κB p65 inflammatory pathway

**Fig. 6:**
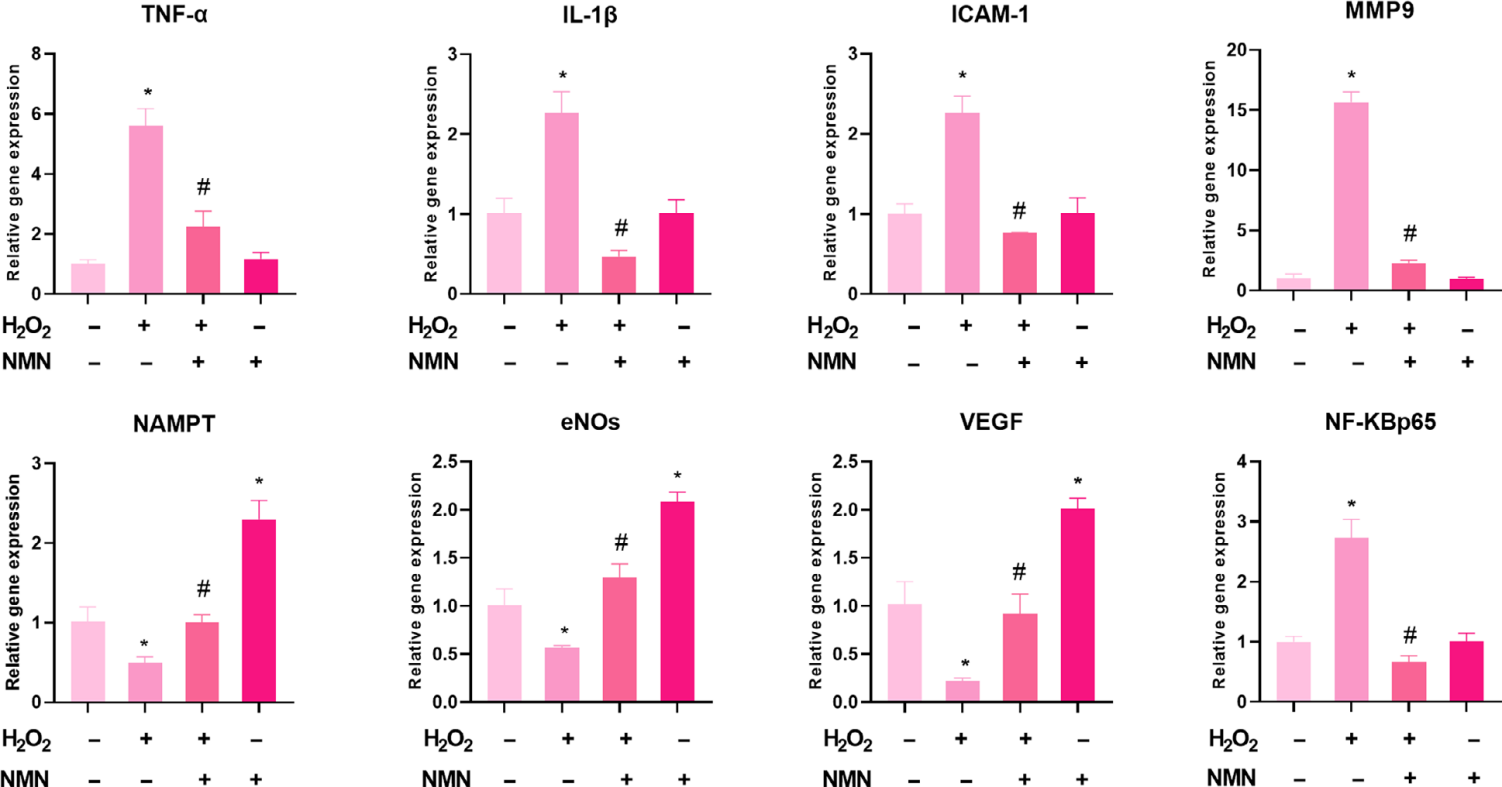
mRNA expression levels of TNF‐α, IL‐1β, ICAM‐1, MMP‐9, NAMPT, eNOs, VEGF and NF‐KBp65 were detected by reverse transcription–quantitative polymerase chain reaction. Results are expressed as means ± SD and are representative of three independent experiments. Data were analysed by one‐way ANOVA with LSD’s test for multiple comparisons. *P < 0.05, vs. control group. #*P* < 0.05, vs. H_2_O_2_ group.

We further examined the effect of NMN on the expression of function‐related factors and NF‐κB inflammatory pathway factors in bEnd.3 cells under hydrogen peroxide stimulation. RT‐qPCR confirmed that NMN inhibited the H_2_O_2_‐induced increase in the NF‐κB inflammatory pathway factors (TNF‐α, IL‐1, ICAM‐1 and MMP9). Moreover, NMN restored NAMPT, eNOS and VEGF expression, which were reduced by H_2_O_2_, to normal levels, and compared with the control group, NMN alone increased the levels of NAMPT, eNOS and VEGF. Moreover, the administration of NMN suppressed the NF‐κBp65 inflammatory pathway and restored endothelial cell‐related functional factors upon H_2_O_2_ exposure (Fig. 6).

### Effects of NMN on the activity of NAMPT and the expression of NF‐κB p65, IL‐1, TNF‐α, MMP9 and ICAM‐1 in oxidative stress‐injured bEnd.3 cells

**Fig. 7:**
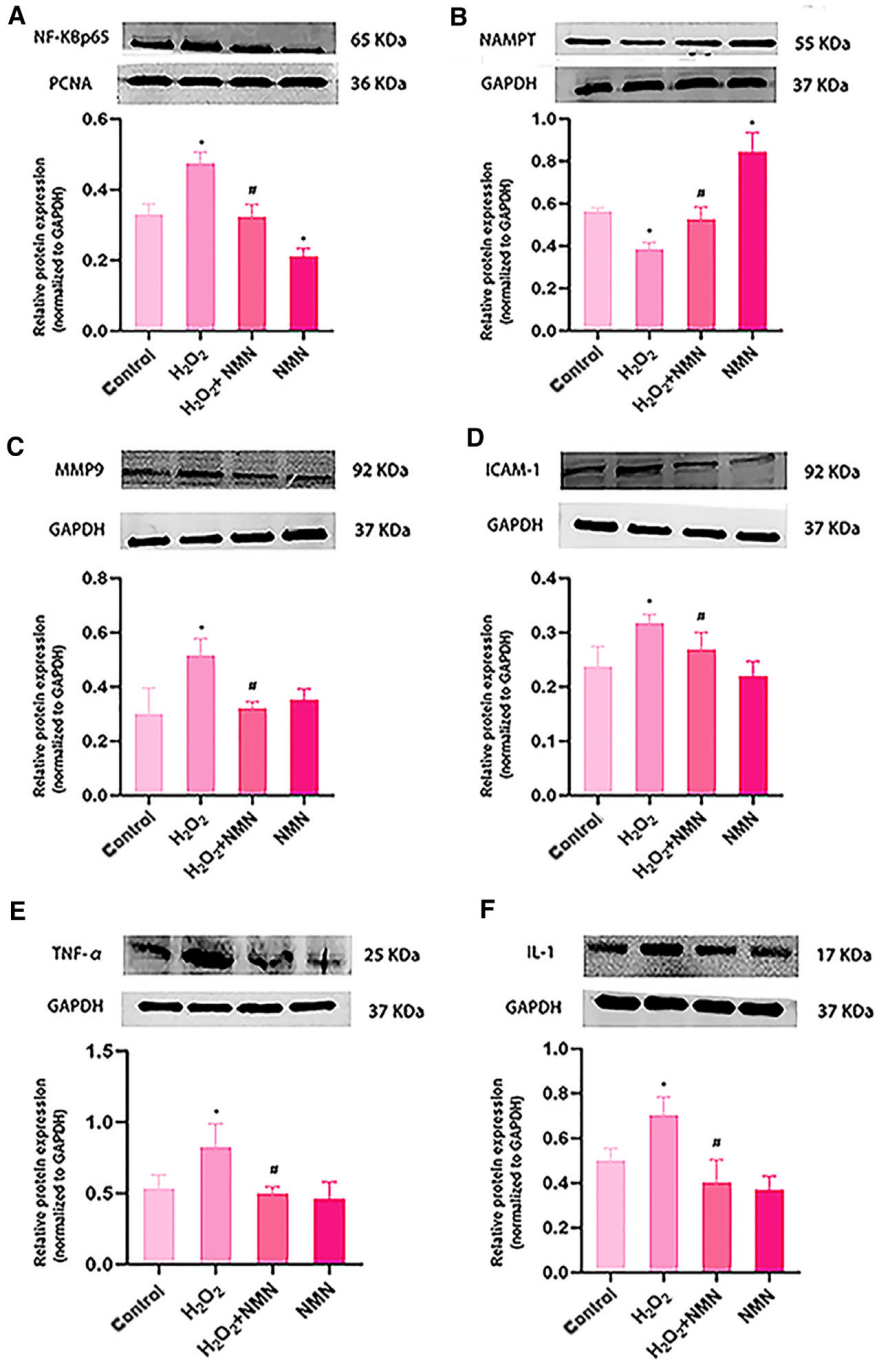
Expression levels of the MMP9, nampt and NF‐KBp65 signalling‐related proteins, TNF‐α, IL‐1β and ICAM‐1 after H_2_O_2_ and NMN treatment in bEnd.3 cells (A–F). Results are expressed as means ± SD and are representative of three independent experiments. Data were analysed by one‐way ANOVA with LSD’s test for multiple comparisons. *P < 0.05 vs the control group ; #*P* < 0.05, vs. H_2_O_2_ group.

NAMPT is a secreted cellular enzyme that participates in stress reactions, such as inflammation, under pathological conditions to avoid apoptosis [20]. Transcription of the proinflammatory cytokines IL‐1β, TNF‐α and ICAM‐1 is mainly regulated by the NF‐κB pathway. Activated IL‐1β and TNF‐α promote endothelial cells to express MMP9 and further exacerbate inflammatory reactions [21, 22]. We further examined the inflammatory signalling pathway to verify the protective effect of NMN on bEnd.3 cells under oxidative stress. As shown in Fig. 6, the expression levels of NF‐κB p65, MMP9, ICAM‐1, TNF‐α and IL‐1β were markedly higher in the H_2_O_2_ groups than in the control group (*P* < 0.05), but the level of NAMPT decreased significantly (*P* < 0.05). Additionally, compared with that of the H_2_O_2_ group, the expression of NF‐κB p65, MMP9, ICAM‐1, TNF‐α and IL‐1β was inhibited in the H_2_O_2_ + NMN group; the protein expression levels of NAMPT notably and significantly increased (Fig. 7 A–F). Compared with the control group, NMN alone significantly increased the protein level of NAMPT and inhibited the expression of NF‐κB p65 (*P* < 0.05). The results of the present study suggested that the enzyme NAMPT and NF‐κB p65 signalling were associated with the protective effects of NMN on H_2_O_2_‐induced bEnd.3 cells.

## Discussion

Diabetic cerebral microangiopathy mainly manifests as damage to the blood–brain barrier, which leads to the dysfunction of vascular endothelial cells. Neurovascular coupling leads to hypoxia‐induced activation of transcription factors, which in turn triggers inflammation and enhances vascular permeability, and studies have shown that the onset of microvascular disease may occur earlier than the onset of type 2 diabetes [23, 24]. Since the blood–brain barrier is mainly composed of vascular endothelial cells, we used bEnd.3 cells to establish a blood–brain barrier model in our experiments. It is generally accepted that oxidative stress and inflammation are key factors that cause endothelial cell dysfunction and increased vascular permeability [25, 26]. Treatment with high concentrations of H_2_O_2_ can cause oxidative stress, inflammation and even apoptosis [27]. Exposure to H_2_O_2_ is a simple and effective in vitro cell model to study oxidative stress‐induced damage in diabetic brain microvascular endothelial cells. Therefore, in the current study, H_2_O_2_ was selected to induce oxidative stress damage in bEnd.3 cells. We first screened the concentration of H_2_O_2_ with CCK‐8 assays, and the results showed that 800 µm H_2_O_2_ had the best inhibitory effect on cell viability after 24 h. The H_2_O_2_ concentration in our experiment was different from the concentration used in other literatures, which might be due to the cell growth status and other external experimental conditions and methods. However, after our repeated verification, 800 µm H_2_O_2_‐induced bEnd.3 cells were an effective and stable model of oxidative stress in our experiments.

Increasing evidence has shown that NMN has beneficial effects on various physiological functions and therapeutic significance in diabetes, obesity, cardiovascular diseases and other disease models [28]. Kiss T et al. [29, 30] suggested that NMN treatment in vivo in ageing rats and in vitro in brain microvascular endothelial cells from aged rats could improve mitochondrial oxidative stress, inflammation, endothelial cell dysfunction and prevent neurovascular coupling (NVC) reactions. In addition, Stefano Tarantini et al. [18] found that NMN supplementation increased endothelial NO‐mediated vasodilation to rescue the NVC response in elderly rats and could reduce mitochondrial oxidative stress and H_2_O_2_ release in elderly brain microvascular endothelial cells. Caton PW et al [31] reported that NMN could correct islet dysfunction induced by inflammatory factors, possibly by inhibiting the expression of genes such as IL‐1β and TNF‐α, and inhibit the nitric oxide enzyme. In the present study, it was suggested that NMN exerted a protective effect against H_2_O_2_‐induced oxidative damage in bEnd.3 cells. In the present study, the results showed that NMN (50, 100 and 300 µm) significantly increased the viability of bEnd.3 cells, indicating that NMN could induce cell survival; then, the effects of NMN on H_2_O_2_‐induced cell viability and apoptosis were measured by CCK‐8 assays, Hoechst 33258 and flow cytometry. The findings at this stage suggested that NMN protected cells from H_2_O_2_‐induced damage by increasing cell vitality. We also measured LDH activity, which further verified the toxic effect of H_2_O_2_ on bEnd.3 cells and the protective effect of NMN on cells. In addition, under the microscope, it was found that the cell volume of the H_2_O_2_ group shrank, the shape was irregular, the cells were significantly reduced and there were many necrotic cells compared with the control group, while the NMN pretreatment group had fuller cell shape and increased adherent cells compared with the H_2_O_2_ group.

Oxidative stress refers to the imbalance in oxidation and antioxidation in the body, which tends to oxidize substances, leading to the inflammatory infiltration of neutrophils, increased secretion of proteases and the production of a large number of key oxidation products. ROS are mainly derived from mitochondria and are an important part of the regulatory transduction pathway; when ROS exceed the antioxidant capacity of the cell, they pose a continuous threat to the cell, resulting in a condition called oxidative stress [32, 33]. Evidence has suggested that NMN treatment can improve mitochondrial oxidative stress and apoptosis in nucleus pulposus cells induced by advanced glycation end products (AGEs) [34]. Wang X et al [35] confirmed that NMN attenuated and eliminated neuronal death and ROS accumulation in rat hippocampal slices treated with Aβ1‐42 oligomers. CAT is a common enzyme that reacts effectively with hydrogen peroxide, and its catalytic activity decomposes hydrogen peroxide into oxygen and water, SOD is an antioxidant enzyme, which plays a central role in neutralizing O2, and when oxidative stress comes, CAT and SOD together form the main line of defence against superoxide radicals and ROS [36]. In order to verify whether endothelial cell damage is related to oxidative stress, the content of ROS and the activity of antioxidant enzymes were measured. In the present study, we confirmed that NMN pretreatment significantly reduced the production of ROS induced by H_2_O_2_ and increased the activity of CAT and SOD. Therefore, NMN could protect bEnd.3 cells from cell damage caused by oxidative stress.

Cytochrome C is also an apoptosis‐promoting protein that can induce apoptosis by activating caspase family, in caspase cascade reaction, apoptosis effector caspase‐3 can be activated by cytochrome C and eventually lead to cell apoptosis [37]. We detected the activity of caspase‐3 and found that compared with the control group, the activity of caspase −3 in bEnd.3 cells was increased by H_2_O_2_ treatment, but decreased by 300 µm NMN treatment, which proved that NMN played an anti‐apoptosis role by blocking the activation of caspase −3.

When oxidative stress occurs, the activation of the transcription factor NF‐κB p65 causes the activation of cellular inflammatory factors, resulting in high expression of IL‐1β and TNF‐α, while the leucocyte adhesion molecule ICAM‐1 and inflammatory factors promote and form a malignant cycle [38]. Regarding vascular endothelial function, studies have confirmed that eNOS signal transduction regulated by VEGF is essential for endothelial cell proliferation and proper angiogenesis [39]; in addition, studies have shown that increased vascular permeability often results in high expression of MMP9 [40]. Our results showed that NMN effectively prevented H_2_O_2_‐induced apoptosis by increasing ΔΨm, downregulating NF‐κB p65, IL‐1β, TNF‐α, ICAM‐1 and MMP9, and upregulating VEGF and eNOS expression. These findings indicate that NMN stabilized endothelial cell function and inhibited apoptosis mediated by the NF‐κB p65 inflammatory pathway, thereby protecting bEnd.3 cells from apoptosis.

NAMPT enzyme is expressed in almost all organs and mainly has two forms: intracellular (iNAMPT) and extracellular (eNAMPT), among which iNAMPT mainly exists in cytoplasm and nucleus [41]. According to previous reports, NAMPT plays a multidirectional role in vascular homeostasis by regulating the functions of endothelial cells, vascular smooth muscle cells, endothelial progenitor cells and perivascular cells [42], and the overexpression of NAMPT can increase proliferation and prolong replication even under high glucose conditions by delaying ageing markers and limiting the accumulation of reactive oxygen species (ROS) [43]. In our study, it was found that the expression levels of NAMPT in bEnd.3 cells were significantly decreased after 800 μm H_2_O_2_‐induced injury and could be reversed by NMN treatment. It is worth noting that when bEnd.3 cells were treated with NMN alone, the gene expression levels of NAMPT, eNOs and VEGF were significantly increased compared with those of the control group, which was consistent with the fact that NMN could increase cell viability, and NMN treatment upregulated NAMPT and downregulated NF‐κB p65 protein expression. The results showed that NMN improved H_2_O_2_‐induced bEnd.3 cell apoptosis not only by inhibiting the NF‐κB p65 inflammatory pathway but also by upregulating the enzyme NAMPT, which may have a certain antagonistic effect.

Taken together, our results demonstrate that NMN is capable of protecting H_2_O_2_‐injured bEnd.3 cells from apoptosis through the regulation of the enzyme NAMPT and the NF‐κB p65 signalling pathway.

## Conflict of interest

The authors declare no conflict of interest.

## Author contribution

ZL and XL designed the experiment and provided the financial support; XD and CL, and YQ conducted the experiment; XD wrote the manuscript; and all authors contributed to data analysis.

## Data Availability

Upon reasonable request, the corresponding author will provide data.
